# Hip fracture systems—European experience

**DOI:** 10.1097/OI9.0000000000000050

**Published:** 2020-03-23

**Authors:** Tim J.S. Chesser, Dominic Inman, Antony Johansen, Alberto Belluati, Carlotta Pari, Achille Contini, Stijn.C. Voeten, Johannes H. Hegeman, Kornelis J. Ponsen, Nuria Montero-Fernández, Alberto Delgado-Martínez, Francisco Chana-Rodríguez

**Affiliations:** a Department of Trauma and Orthopaedics, Southmead Hospital, Bristol; b Department of Trauma and Orthopaedics, Northumbria Healthcare NHS Trust; c Department of Orthogeriatrics, University Hospital of Wales, Cardiff, UK; d Department of Trauma and Orthopaedics, Hospital Santa Maria delle Croci, Ravenna, Italy; e Department of Trauma and Orthopaedics, Hospital Santa Maria di Loreto, Naples, Italy; f Department of Trauma Surgery, Leiden University Medical Center, and Dutch Institute for Clinical Auditing, Leiden; g Department of Surgery, Ziekenhuisgroep Twente, Almelo-Hengelo; h Department of Surgery, Noordwest Ziekenhuisgroep, Alkmaar, The Netherlands; i Geriatrics Department, General University Hospital Gregorio Marañón, Madrid; j Orthopaedic Department, General University Hospital of Jaen, Jaen; k Orthopaedic Department, General University Hospital Gregorio Marañón, Madrid, Spain

**Keywords:** hip fracture care, national systems, systems of care

## Abstract

European countries have established health care systems but are struggling with the increasing rise of fragility fractures in their aging population. In trying to address this significant burden, countries are establishing national guidelines and standards, focusing on hip fractures, which represent the significant cost for this patient group. This has evolved with the establishment of national audits and guidelines. Reports from 4 European countries (England, Italy, Netherlands, and Spain) are presented. All nations have identified both deficiencies in their systems, and protocols to improve these deficiences. When standards are introduced, there has been evidence of improved results. Significantly more work is needed to understand the key components of the systems and pathways, and efforts to study and standardize care are ongoing.

## 1 Introduction

Hip fracture care and systems of care for fragility fractures are developing in Europe. The incidence has been slowly rising over the last 20 years, but now stabilizing to approximately 1 fracture per 1000 patients in the majority of countries. This is despite the aging population and may reflect the increased use of prevention with bone health and falls prevention. The care costs associated with a hip fracture create a significant burden of heath care resource (up to 1.5% of total health care budgets). In countries with established health care systems, there is a trend of setting up national audits and writing standards to try to measure and improve the treatment of these patients. Below are reports from England, Italy, Netherlands, and Spain, who are all at different phases of this process.

## 2 Hip fracture care and national system in England

England has delivered a major system change in hip fracture care over the last 10 years. The health system is tax-funded and freely accessed at the point of delivery. For a number of years, there was recognition of a disjointed approach to hip fracture care, with numerous publications trying to address the issues of multidisciplinary and coordinated care, yet the results in terms of length of stay and mortality remained unchanged. For many it was considered a medical problem as the majority of the surgery required could be performed by junior orthopaedic staff, often on unsupported emergency lists, with junior anesthetists. Complications were often put down to the complex patient group, who present with significant medical comorbidities. The patient group, like others who present with emergencies, had no voice, with no public outcry of the poor standards of care often given. There has been a paradigm shift in how these patients are cared for, managed, and measured. They are now a benchmark of how hospitals treat emergency admissions, because if there is infrastructure to treat hip fractures well with the huge number of disciplines involved, it is likely other emergency admissions will receive the same level of care.

This change was initiated by 2 major projects. First, realizing that one is unable to judge something unless one measures it, and second, unless one creates standards, one can never audit the care. From this, the National Hip Fracture Database (NHFD) was born in 2007, in the first few years supported by enthusiastic hospitals, and now by every hospital in England.^[1]^ Over 650,000 cases have been added since 2007, with now 176 hospitals, contributing over 5000 patients per month (approximately 55,000 a year). The database is centrally funded via government but is run independently. The initial standards were written in a joint publication between the British Orthopaedic Association and the British Geriatric Society in the form a “Blue Book” titled “The Care of Patients with a Fragility Fracture,”^[2]^ which was the initial core standards collected by the NHFD. This subsequently was superseded by National Institute of Health and Social Care (NICE) hip fracture guidelines in 2011.^[3]^

In 2010, a Best Practice Tariff (BPT) was created to financially incentivize improved care.^[4]^ This was based on a set of processes or surrogates, which was felt if delivered, would lead to improvements in outcomes. The original criteria included the requirement for a multidisciplinary pathway, orthogeriatric review within 72 hours, bone health and falls assessment, surgery within 36 hours, and submission of data to the NHFD. The initial “carrot” (which was afforded by dropping the national tariff) was £440 ($570) for each individual patient achieving the criteria. Due to its success, the BPT has increased to £1335($1750) with additional criteria of preoperative cognitive assessment, postoperative delirium screening, nutritional assessment, and early mobilization targets added. In the first year, just over 20% of patients were achieving the criteria to obtain the tariff—10 years later this has risen to 60%.

The NICE guidance was challenged to provide standardization in areas where there remained a huge variation in practice and reported on all aspects of the pathway from initial assessment, timing of surgery, standardizations of surgical procedures, mobilization, and rehabilitation. From the NICE guidance have come the NICE quality standards, of which the majority can be monitored by the NHFD.

The guidelines include that patients should be managed with combined orthopaedic and orthogeriatric care in a hip fracture programme. The national guideline is that all patients are seen preoperatively, yet due to difficulties in accessing orthogeriatricians at weekends, the target is that all patients are reviewed within 72 hours from admission.

For patients taking anticoagulants, which currently reflects about 20% of the population, all hospitals have agreed protocols for patients taking warfarin (coumadin), which usually is the injection of Vitamin K in the emergency department and subsequent regular testing for the International Normalised Ratio (INR). With the newer direct oral anticoagulants, the majority of hospitals agree to proceed from 24 hours after the last dose, as there is limited evidence this does not lead to major hemorrhage.^[5]^ The national standard for time to surgery is within 36 hours of presentation. This is now achieved in 70% of the patients presenting. The commonest reason for delay is lack of theater availability.

Regarding the type of anesthesia, NICE guidance suggested no difference in outcome between general and regional anesthesia in this population, so both are used, with presently 58% of patients receiving a general anesthetic and 42% a spinal. Efforts have been concentrated on reducing intraoperative hypotension and using additional nerve blocks to aid analgesia. Protocols were introduced for surgical treatments of the common fractures. NICE guidance advised replacement arthroplasty for displaced subcapital fractures, with the use of total hip replacement for those without cognitive impairment, medically fit, and walking outdoors with no more than a stick. The current rate of cemented arthroplasty is up to 90%, yet less than 40% of those eligible for a total hip replacement receive one. For undisplaced subcapital fractures, due to lack of evidence, no recommendations could be given. For the treatment of trochanteric fractures, the NICE guidance recommended sliding hip screws (SHSs) for A1 and A2 fractures (achieved in 75%) due to the equivocal clinical results of extramedullary plates compared with intramedullary nails, but a cheaper cost with SHS. For A3 fractures, there was no recommendation due to lack of evidence and for subtrochanteric fractures intramedullary nails were advised.

It was mandated that all hip fracture patients should be allowed to fully bear weight without restriction in the immediate postoperative period. National guidance states that they should be mobilized on the day or day after surgery and daily there afterward, with physiotherapy support and assessment. All patients should undergo screening and subsequently be treated appropriately regarding their bone health and falls prevention to prevent future fractures. There remains very little follow-up of these patients outside research. Attempted follow-up at 1 year proved to be very difficult, so efforts are made to perform telephone follow-up at 4 months.

What have the changes led to? All hospitals have changed their practice. Hip fractures are both on local and national agendas. The patients now receive urgent care (often prioritized on Consultant led Trauma lists, with senior surgeons and anethetists) with both early orthogeriatric input and multidisciplinary rehabilitation. There has been standardization of surgical procedures, and the hospitals are rewarded with more income to invest in services. The information of performance (through the NHFD), including mortality, has been published on the Internet with open access to all, in real time, with annual reports highlighting key issues. Those who struggle to deliver or maintain standards are offered independent multidisciplinary reviews to try to address local issues, and the standards continue to rise. It was previously quoted that treating hip fractures well was cheaper than treating them badly, and this has now been proven. In just a few years, the mortality associated with a hip fracture has dropped by up to 40%, and the cost of treating these patients has reduced rather than increased. Better care is not always more expensive care.

## 3 Hip fracture care and national system in Italy

At present, Italy lacks a National Hip Fracture Care System, due to the many different local practices. However, one approach is to identify best practice guidelines followed in our Regional Care System (Area Vasta Romagna) as a model and expand it to other institutions in the near future. Patients admitted to emergency departments with the suspicions of a hip or femoral fracture are immediately sent to the radiology department to undergo radiographs of the affected hip, and CT scan in those cases where the diagnosis is in doubt. If a hip fracture is confirmed, chest radiographs, electrocardiogram, preoperative blood tests are performed, and the patient is sent to the Orthopaedic Department within 4 hours from the admission.

The pain is immediately assessed using the numerical rating scale and periodically reassessed. For noncooperative patients, the Pain Assessment in Advanced Dementia scale is used. Pain management consists of an intravenous paracetamol injection as first-line drug. Opioids can be added as second-line drugs, depending on the perceived pain.

The multidisciplinary preoperative evaluation team includes the orthopaedist, the anesthetist, and the geriatrician, aiming to perform the surgery within 48 hours from the trauma, ideally within the first 24 hours. Particular attention is paid to all those clinical factors that could cause a delay in the surgical treatment. Major factors that are required to be corrected before the surgery, and that legitimize a delay, are as follows:^[6]^

1. Significant blood pressure alteration, with a systolic pressure less than 90 mm Hg
2. Severe heart arrhythmia: atrial fibrillation or supra-ventricular tachycardia ≥121 bpm, bradycardia ≤45 bpm, third-degree atrioventricular block
3. Infections/acute pneumonia with body temperature ≤35°C or ≥38.5°C
4. Acute myocardial ischemia
5. Respiratory failure/pulmonary edema
6. Severe electrolyte disorders: Na < 125 or > 155 mEq/L, K < 2.5 or > 6.1 mEq/L, HCO_3_ <18 or > 36 mEq/L
7. Uncontrolled diabetes with glycemia ≥600 mg/dL
8. Severe anemia: Hb ≤ 7.5 g/dL

The chance of an early surgical treatment is also related to the operating room availability.

The increased use of antithrombotic therapy in the older population is another factor that can lead to a delayed treatment. According to the literature,^[7,8]^ a target of preoperative INR value less than 1.5 is aimed for, following the schema reported below:

1. Anticoagulation therapy interruption at the patient arrival
2. If the INR value is less than 1.5, surgical intervention may proceed
3. If the INR value is more than 1.5, vitamin K intravenous injection (1–5 mg). INR check in 6–12 hours; if value is still more than 1.5, vitamin K injection can be repeated
4. Low-molecular-weight heparin treatment is not initiated until an INR value less than 2 is achieved
5. Surgical treatment within 24 hours after recoagulation
6. For patients taking newer anticoagulants such as dabigatran, rivaroxaban, and apixaban, there is no reversal therapy, and a prudent time of approximately 48 hours between the last dose and the surgery is recommended
7. The use of fresh-frozen plasma is indicated only in cases of massive bleeding that require the immediate recoagulation

Our target is to be able to proceed to the surgical treatment within 48 hours from the trauma and to obtain a stable fixation, which allows us to start the rehabilitation programme the first day after the surgery. We perform the following:

1. Intramedullary nail fixation for the extra-capsular fractures
2. Internal fixation with screws for the nondisplaced intracapsular fractures in younger patients in which the life expectancy is higher than prostheses duration
3. Uncemented total hip arthroplasty for displaced intracapsular fractures in patients less than 80 years old
4. Uncemented hemi-arthroplasty for displaced and nondisplaced intracapsular fractures in patients over 80 years old
5. Uncemented total hip arthroplasty using dual-mobility cups for intracapsular fractures in patients less than 80 years old with neurological diseases, and in elderly patients in which hemi-arthroplasty is not indicated (pre-existing advanced arthritis of the hip, dysplasia, recurrent hemi-arthroplasty dislocation, failure of osteosynthesis devices previously implanted)
6. Uncemented implants are the standard in Italy

During postoperative care, the role of the geriatrician becomes indispensable to prevent the onset of minor/major medical complications. If complications occur, they will need to be promptly managed to restrict the short/long-term mortality rate. The role of the physiotherapist becomes essential to maximize the patients’ clinical outcomes, with efforts to educate patients and their relatives to the importance of early mobilization and new falls prevention.

## 4 Hip fracture care and national system in the Netherlands

In the Netherlands, approximately 18,500 hip fracture patients were treated in 81 hip fracture operating hospitals in 2017, with the number of hip fracture patients per hospital ranging from 9 to 717.^[9]^

### 4.1 Hip fracture guidelines

Two evidence-based Dutch guidelines form the basis for optimal hip fracture care. The “Guideline Proximal Femur Fracture,” most recently updated in 2016, was developed by the Dutch Association of Surgeons (NVvH) and the Dutch Orthopaedic Association (NOV) and affirmed by the Dutch Geriatric Society (NVKG) and the Dutch Society of Anesthesiologists (NVA).^[10]^ This guideline focuses on the surgical treatment of all hip fracture patients. The “Guideline Treatment of Frail Elderly During Surgical Procedures,” first published in 2016, was developed by the NVKG and affirmed by the national medical societies representing the caretakers of this patient population.^[11]^ This guideline focuses on how the multidisciplinary hip fracture care should be organized and the treatment of the frail elderly patients.

### 4.2 Quality of care regulators

Two governmental institutions supervise the quality of hip fracture care: the Dutch National Healthcare Institute (ZINL) and the Health and Youth Care Inspectorate (IGJ). Each year quality indicators at hospital level are defined, and hospitals are obliged to deliver their results on the quality indicators. The results are published in May of the following year. Underperforming hospitals (i.e., hospitals outside the confidence interval in the funnel plots) are being evaluated, and need to present improvement strategies for the coming year(s) to the responsible institutions.

### 4.3 Orthogeriatric comanagement

Orthogeriatric comanagement during hospital stay, starting preoperatively, should be available for all patients from 70 years of age, as well as for high-risk patients younger than 70 (e.g., comorbidity, malnourished).^[3]^ The IGJ has used this parameter as a quality indicator in 2017, with on average 80% of the patients receiving orthogeriatric comanagement.^[12]^

### 4.4 Time to surgery

Hip fracture patients need to undergo surgery on the day of admission or the following day.^[9,10]^ Legitimate reasons for delay of the operation have been defined: anemia, anticoagulation, volume depletion, electrolyte imbalance, uncontrolled diabetes, uncontrolled heart failure, correctable cardiac arrhythmia or ischemia, pneumonia, and exacerbation of chronic obstructive pulmonary disease.^[11]^ In general, the majority of these contra-indications can be optimized within 24 hours, avoiding further delay. The ZINL used the median time to operation as a quality indicator in 2017. For American Society of Anesthesiologists (ASA) I-II patients, this parameter was 18 hours (interquartile range 7–23 hours) and for ASA III-IV patients, 21 hours (interquartile range 12–27 hours).^[13]^ This suggests that implementation of this part of the guidelines is well established.

### 4.5 Protocols for anesthesia and for patients on anticoagulation

Upon arrival at the emergency department, the assessment of the pain scores should be started. This assessment is repeated 30 minutes after administering the initial analgesia and subsequently every hour until arrival at the ward. On the ward, pain scores then need to be assessed 3 times a day.^[10]^ The choice of drugs for the pain treatment is protocolized, the use of nerve blocks is facultative and might be helpful, although the guideline states that more evidence is needed.^[10]^ Intraoperative anesthesia is either general or spinal anesthesia, and an intraoperative nerve block is considered to reduce postoperative use of opioids. In 2017, 30% of patients received general anesthesia, 45% spinal anesthesia, 10% a combination of general and spinal anesthesia, and at that time, 15% of the data on perioperative anesthesia was missing.^[13]^ In the postoperative setting, the pain scores and pain medication follow the preoperative recommendations.

Anticoagulation is a legitimate reason for delay. For patients on warfarin, the INR needs to be <1.8 for spinal anesthesia; direct oral anticoagulants either postpone the operation for 48 hours or anticoagulation needs to be reversed.

### 4.6 Protocols for fracture treatment

For nondisplaced femoral neck fractures, it is recommended to use either an SHS or cancellous screws, based on surgeon preference.^[10]^ The optimal treatment strategy for displaced femoral neck fractures depends on the patient characteristics. For fit patients (ASA I–II and 18–80 years), internal fixation can be considered, while for relatively fit patients in the older category (ASA I–II, 61–80 years, no cognitive impairment and outdoor mobility using not more than one aid) total hip replacement is advised. For patients over 60 years who do not directly fit in the previous 2 categories, hemiarthroplasty can be an option after discussing all options with either the patient and/or the family (shared decision making).^[10]^ For trochanteric fractures 31-A.1, the SHS is recommended, and for 31-A.2 and A.3 both an SHS and intramedullary fixation are options.^[10]^ Subtrochanteric fractures should be fixed with a long intramedullary nail.^[10]^

### 4.7 Protocols for rehabilitation

After the operation, the rehabilitation process should be organized on a multidisciplinary basis and individual targets should be set in order to restore patient mobility and independence as quickly as possible to the prefracture level.^[3]^ Starting at day 1 after the operation, patients need to be mobilized at least once a day, full weightbearing (as tolerated), with a physiotherapist.^[10]^ After discharge, the multidisciplinary rehabilitation needs to be continued, with physiotherapy, mental health screening, fall prevention, and osteoporosis screening.^[10,11]^

### 4.8 The Dutch hip fracture audit

In April 2016, the nationwide Dutch Hip Fracture Audit (DHFA) was launched. The DHFA aims to improve the overall quality of hip fracture care by providing hospitals insight into their performance. Hospitals can use this feedback to initiate targeted interventions and to improve their quality of hip fracture care.^[13]^ The multidisciplinary clinical audit board, which consists of doctors of 4 different medical associations (trauma surgeons, orthopaedic surgeons, geriatricians, and internists), is responsible for the content of the audit.^[13]^

Participation in the DHFA by hospitals is not obligatory, and there is no financial gain or financial support for participating hospitals. Besides the fact that participation in the DHFA leads to feedback at the hospital level, it also has the advantage that all quality indicators can be directly calculated from the DHFA registry and be delivered to the 2 governmental institutions. Therefore, using the DHFA prevents hospitals from recording the same data for different institutions in different databases. The DHFA itself is accommodated within the Dutch Institute for Clinical Auditing, an organization that facilitates nationwide audits and is funded by the health care insurance companies.

In the DHFA, 45 items are prospectively recorded in a web-based survey.^[13]^ Besides clinical data, the functional scores (mobility score and Katz-6 ADL score) at 3 months following surgery are also collected. In 2017, the first full year of the registration, the proportion of completed variables of the clinical data was already good with 91%.^[13]^ However, the functional scores at 3 months were only achieved in 26% of all cases.^[14]^ As there are no mandatory protocols for follow-up of hip fracture patients, it appears to be difficult for hospitals to collect the functional scores. Nevertheless, the ZINL, IGJ, and health insurance companies want to have insight in these different parameters and outcomes. So, sooner rather than later it can be expected that the registry will achieve significantly higher scores of completeness in the follow-up section.

In 2017, the DHFA quality indicators still showed a considerable practice variance between hospitals in both orthogeriatric comanagement and time to surgery.^[13]^ In line with other hip fracture audits, it can be expected that reporting these results will lead to improvements in hip fracture care in the Netherlands in the coming years.

## 5 Hip fracture care and national system in Spain

### 5.1 Funding

Health care for ordinary illnesses and nonoccupational accidents in Spain is a noncontributory benefit and is funded by taxes. Access to public health services is guaranteed by Article 43 of the Spanish Constitution of 1978, which establishes the right to health care for all citizens. The National Health System has 315 hospitals, equipped with 105,505 beds.

According to the National Statistics Institute, the population of Spain registered on the July 1, 2018 census was 46,733,038 inhabitants. This population had a life expectancy at birth of 78.6 years for men and 84.9 years for women in 2009.^[15]^ Statistical data from the Spanish Ministry of Health show an incidence of elderly hip fractures of 104 cases per 100,000 inhabitants in 2008. In 2008, 45,000 hospital discharges related to hip fractures occurred, of which 85% were elderly (≥75 years), with an average female/male ratio of 3.5.^[16]^

Hip fracture care means a high cost for the Spanish health system. Mean total cost during the first year after a hip fracture was calculated at €9690 (95% CI: 9184–10,197) in women and €9019 (8079–9958) in men. The main cost determinant was initial hospitalization (€7067 and €7196 in women and men), followed by ambulatory care and home care.^[17]^

### 5.2 Guidelines

There are no official national guidelines available for the management of hip fractures, but some regions have developed their own guides. However, there are several working groups of different medical societies (Spanish Society of Geriatrics and Spanish Society of Orthopaedics) that establish recommendations based on other international guidelines. In 2017, the Spanish hip fracture registry was initiated as a multicentre, observational, descriptive study of the epidemiological, clinical, and care characteristics to describe the existing situation and establish possible points of improvement throughout Spain.^[18]^

### 5.3 Operative care

The most common model in Spain consists of the collaboration between geriatrics/internal medicine and orthopaedic traumatology to assist in the acute care phase. Many hospitals already have orthogeriatric units with shared responsibility between geriatrics and traumatology. Seven percent of patients are just evaluated by the orthopaedic surgeon during the hospital stay. In addition to the surgeons, in most cases geriatricians also evaluated these patients (80%). Thirteen percent are followed by internists.^[18]^

### 5.4 Anticoagulation

According to the current guidelines and protocols, patients taking 100 mg/d of aspirin are not contraindicated for surgical intervention, while those taking 300 mg/d of aspirin are placed on alternative medications. Clopidogrel is discontinued and patients wait 5 days before the intervention. Acenocumarol (Coumadin) is usually reversed with vitamin K at admission. Oral direct anticoagulants are discontinued before surgery according to their half-life. Postoperative anemia is the most frequent complication, with 53% of the fractures requiring a transfusion. Eighty percent of physicians transfuse with Hb < 8 g/dl, except if there is cardiorespiratory or cerebrovascular pathology associated. Tranexamic acid is starting to be used but not widespread yet. Low molecular weight heparins are used to prevent deep vein thrombosis in nearly all patients, and tromboprophylaxis is extended during 35 days after surgery.^[18]^

### 5.5 Timing

Currently, performing surgery within the first 48 hours is considered a quality standard, but a goal that has not been achieved in most of the Spanish centers (less than 30% of the centers attain the standard). The mean surgical delay in the Spanish registry is approximately 3 days (76 hours), although it varies between 1 day and 6 days depending on the hospital. The cost per day of hospital stay has been estimated at 1000 €, so a delay of 1 day for hip surgery has an approximate cost of 1800 €.^[19]^ Each day of delay in hip surgery leads to an extension of 1.8 days in the hospital stay. The delay in surgery was due to logistical problems in 44%, anticoagulants or antiplatelet agents in 12%, and clinical instability in 7%.^[18]^

### 5.6 Anesthesia

According to the ASA physical status classification system, 67% of these patients are ASA > 2. Neuraxial anesthesia is the most common technique (88%). It is recommended over general anesthesia, as it has been shown to reduce time, costs, complications, and mortality. Fifty seven percent of hospitals in Madrid have a protocol for pain management in hip fractures. The most used painkillers are paracetamol (93%), dipyrone (57%), and non-steroidal-anti-inflammatory drugs (35%). If pain control is insufficient, opioids are used (usually tramadol).^[18]^

### 5.7 Fractures

According to the Spanish Hip Fracture Registry, 11% were nondisplaced intracapsular fractures, 28.3% were displaced intracapsular fractures, 52% were pertrochanteric fractures, and 7.2% were subtrochanteric fractures. Ninety five percent of patients underwent surgery. The implants used were 2% cannulated screws, 57% intramedullary nails, 1% dynamic hip screws, 37% hemiarthroplasties, and 3% total hip arthroplasties. Two percent of patients developed a complication that required additional surgery within the first month (0.4% reduction of dislocated arthroplasty, wound debridement 0.5%). In-hospital mortality reached 5.3%, and 1-month mortality reached 7.6% for hip fracture patients.^[18]^

### 5.8 Rehabilitation

Most patients receive rehabilitation during their hospital stay (75%). If possible, patients are seated in the first 24 hours postoperatively and start walking between 36 and 48 hours. Immediate unlimited weightbearing is allowed in nearly 90% of hip fracture patients. Eighty percent were ambulatory prior to the fracture, but only 13% ambulated independently at discharge, and 58% after 1 month. After discharge, 40% required a medium-term functional recovery unit.^[18]^ The mean hospital stay is 11 days (6–20).

### 5.9 Prevention

In 2014, a national protocol for the prevention of falls in elderly patients was published. Bone health screening was not performed for primary prevention as a routine procedure. For the pharmacological treatment of fragility hip fractures, 5% used antiresorptive or osteoinductive drugs, 13% calcium, and 17% vitamin D prior to the fracture. After hospital discharge, 37% were prescribed antiresorptive or anabolic drugs, 50% calcium, and 70% vitamin D(4).

### 5.10 Follow-up

The Spanish Hip Fracture Registry is a multicenter, observational, prospective audit endorsed by over 20 regional and national scientific societies. Started in January 2017, the registry includes all patients 75 years or older admitted for hip fractures in any of the participating centers, followed for 30 days. Registry members enter data on a voluntary basis. Donations from industry sponsorship have allowed for the registry to hire statisticians and administrative personnel for its maintenance. The registry results are published every year.^[18]^

### 5.11 Improvements

In recent years, collaboration with geriatricians has been established, improving postoperative complications, average hospital stay, functional recovery, and costs. Another target is to perform surgery within the first 48 hours, as this figure is a quality indicator in these patients.

## 6 Conclusion

In Spain, hip fractures in the elderly represent a public health problem due to their prevalence, consequences for patients and relatives, and total cost in a society that advocates universal health coverage.

## 7 Summary

All nations have identified both deficiencies in systems and protocols to improve these systems. In Europe, national audits and standards are increasingly being introduced and have shown to improve results. Significantly more work is needed to understand the key components of the systems and pathways, and efforts to study and standardize care are ongoing.
